# Supplementary material for: The interactome of intact mitochondria by cross-linking mass spectrometry provides evidence for coexisting respiratory supercomplexes
Source: Mol Cell Proteomics. 2017 Dec 8;17(2):216–32. doi: 10.1074/mcp.RA117.000470 (PMC5795388; doi:10.1074/mcp.RA117.000470)
Supplement: Supplemental Data [file supp_RA117.000470_133922_0_supp_23978_fzffwf.zip › spectra_annotation/mito_spectra_annotation/402-1-12-1-2-1.pdf]

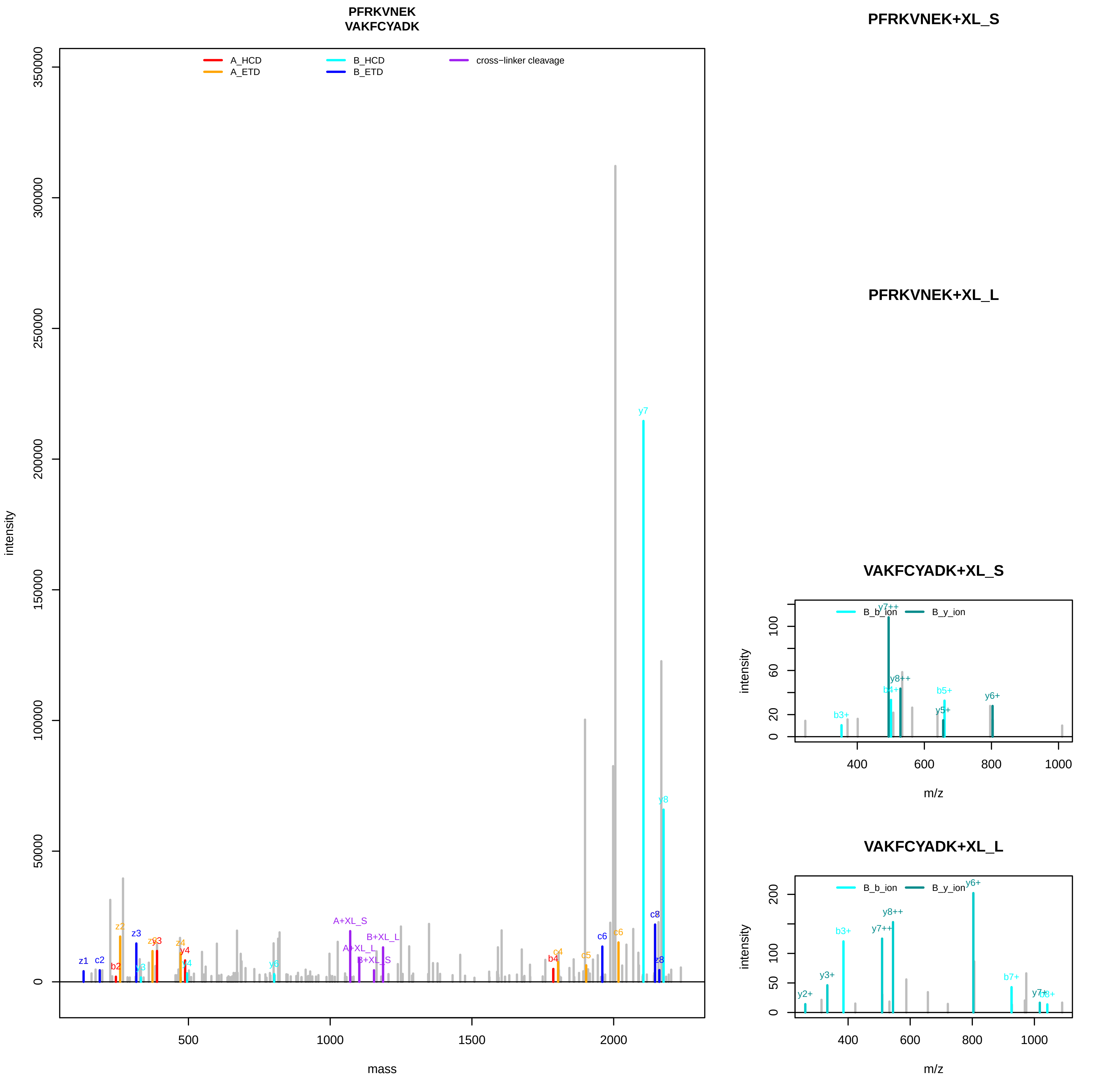

PFRKVNEK
VAKFCYADK
A_HCD
A_ETD
B_HCD
B_ETD
cross−linker cleavage
intensity
mass
0
50000
100000
150000
200000
250000
300000
350000
500
1000
1500
2000
y7
y8
z1
c2
b2
z2
z3
y3
z4
y4
A+XL_S
B+XL_L
A+XL_L
B+XL_S
b4
c4
c5
c6
c8
z8
PFRKVNEK+XL_S
PFRKVNEK+XL_L
VAKFCYADK+XL_S
B_b_ion
B_y_ion
y7++
y8++
b4+
b5+
y5+
y6+
b3+
m/z
400
600
800
1000
0
20
60
100
VAKFCYADK+XL_L
y6+
y8++
y7++
b3+
y3+
y2+
b7+
y7+
b8+
0
50
100
200
